# Vitamin-D-Supplementation bei Depression – sinnvoll, evidenzbasiert oder doch leitliniengerecht?

**DOI:** 10.1007/s00115-026-02000-2

**Published:** 2026-07-31

**Authors:** Astrid Röh, Ramona Hiltensperger, Michael Schepp, Theresa Halms, Alkomiet Hasan, Annabel Sandra Mueller-Stierlin

**Affiliations:** 1 Klinik für Psychiatrie, Psychotherapie und Psychosomatik, Medizinische Fakultät, Universität Augsburg, Bezirkskliniken Schwaben, Bezirkskrankenhaus Augsburg, Geschwister-Schönert-Straße 1, 86156 Augsburg, Deutschland; 2 Deutsches Zentrum für psychische Gesundheit (DZPG), Standort München/Augsburg, Augsburg, Deutschland; 3 Institut für Epidemiologie und Medizinische Biometrie, Universität Ulm, Ulm, Deutschland

## Hintergrund

Wenn im Herbst die Tage kürzer und die Sonnenstunden seltener werden, nimmt die endogene Vitamin-D-Synthese ab und die Serumspiegel sinken kontinuierlich. Vitamin D wird hauptsächlich in der Haut durch UV-B-Strahlung aus 7‑Dehydrocholesterin gebildet. In nördlichen Breiten, wie in Mitteleuropa, reicht die UV-B-Intensität in den Herbst- und Wintermonaten jedoch nicht aus, um eine ausreichende eigene Vitamin-D-Produktion aufrechtzuerhalten. Dadurch liegen die Serumwerte in dieser Jahreszeit häufig in einem Bereich, der als unzureichende Versorgung gewertet wird. Weitere Einflussfaktoren auf den Vitamin-D-Status beinhalten beispielsweise die Hautpigmentierung, Körpergewicht, Körperbedeckung, Aufenthaltsdauer im Freien sowie Alter, aber auch Ernährungsgewohnheiten [1, 8]. Vor diesem Hintergrund stellt der niedrige Vitamin-D-Status in der mitteleuropäischen Bevölkerung, insbesondere im Winter, ein relevantes Gesundheitsproblem dar, das mit verschiedenen Krankheiten, einschließlich psychischer Erkrankungen, assoziiert sein kann [4].

Für Menschen mit Depressionen kann ein niedriger Vitamin-D-Spiegel eine zusätzliche Belastung darstellen. Neben der eigentlichen psychischen Erkrankung können sich die Folgen eines unzureichenden Vitamin-D-Status ungünstig auf Stimmung, Energie und allgemeines Wohlbefinden auswirken. Unter diesem Aspekt erscheint eine Vitamin-D-Supplementation zunächst als naheliegende, unterstützende Maßnahme.

## Kontroverse der Supplementation in Leitlinien und Praxis

Die Deutsche Gesellschaft für Ernährung (DGE; [3]) empfiehlt eine Bestimmung der Serumwerte und gegebenenfalls anschließende Supplementation insbesondere bei Risikogruppen (Personen höheren Alters, Personen, die sich bei Sonnenschein kaum oder gar nicht im Freien aufhalten oder Personen mit dunkler Hautfarbe, mobilitätseingeschränkte, chronisch kranke und pflegebedürftige ältere Menschen in Pflegeeinrichtungen sowie Säuglinge im 1. Lebensjahr). Sie betonen die vor allem im Winter häufig unzureichende endogene Synthese, was bei 60 % der Bundesbürger dazu führt, dass die wünschenswerte Blutkonzentration des Markers 25-Hydroxyvitamin‑D von 50 nmol/l nicht erreicht wird. Gleichzeitig warnt das Bundesinstitut für Risikobewertung aufgrund gesundheitlicher Risiken vor einer Supplementation mit hohen Dosen (z. B. 500 μg alle 20 Tage), insbesondere bei Personen, die bereits sehr gut mit Vitamin D versorgt sind.

Die internationale Leitlinie der World Federation of Societies of Biological Psychiatry (WFSBP) und des Canadian Network for Mood and Anxiety Treatments (CANMAT; 2022) spricht auf Basis einer Metaanalyse (k = 27, *n* = 7651) sowie zweier randomisierter kontrollierter Studien (RCTs) eine schwache Empfehlung für den Einsatz von Vitamin D bei Major-Depression aus [7]. Methodisch wird die Evidenz mit Level A (mindestens zwei RCTs oder einer Metaanalyse) klassifiziert – also als formal hochwertig –, auch wenn die Leitliniengruppe selbst auf Limitationen der zugrunde liegenden Studien hinweist (unter anderem die hohe Heterogenität der Population) und damit die klinische Empfehlung als schwach einzustufen ist. Zudem erfordert die Dynamik der Evidenzlage durch kontinuierlich erscheinende, teils heterogene Metaanalysen [6, 9] eine fortwährende Aktualisierung. Dies unterstreicht die Notwendigkeit, den Einsatz von Vitamin D stets populationsspezifisch und diagnostisch gestützt zu bewerten.

So vertreten die Autoren der Nationalen Versorgungsleitlinie (NVL) „Unipolare Depression“ (2022; [2]) eine zurückhaltende Haltung: Eine Substitution sollte wenn überhaupt bei nachgewiesenem Mangel erfolgen. Der klinische Nutzen der Supplementierung in der therapeutischen Behandlung von Depressionen wird als unklar bewertet. Neben unzureichender Evidenz werden mögliche Risiken erwähnt, darunter die Gefahr, leitliniengerechte Therapien zu vernachlässigen, sowie einer Fehlwahrnehmung, Nahrungsergänzungen könnten eine ausgewogene Ernährung ersetzen. Die NVL empfiehlt vielmehr eine ausgewogene Ernährung der Patientinnen und Patienten zu fördern, doch in der Praxis fehlen meist die Ressourcen und Konzepte, um diese Empfehlung umzusetzen.

Die Negativ-Empfehlung der NVL, die lautet „Wenn kein Mangel vorliegt, sollen Nahrungsergänzungsmittel nicht empfohlen werden“ (Empfehlungsgrad A), ist jedoch als Aufforderung zum diagnostikgestützten Vorgehen zu verstehen, nicht jedoch als pauschale Ablehnung der Supplementation.

Hinzu kommt jedoch ein praktisches Problem: Trotz der Vulnerabilität für Mangelzuständige, wird die erforderliche Laborbestimmung zum Nachweis eines Vitamin-D-Mangels bei Menschen mit Depressionen in der psychiatrischen Versorgung bisher nicht systematisch durchgeführt. Dies könnte daran liegen, dass die Kostenübernahme für diese Laboruntersuchung durch die Kassenärztlichen Vereinigungen (KVen) aktuell nicht einheitlich geregelt ist. Die Entscheidung zur Indikationsstellung liegt im ärztlichen Ermessen und muss ausreichend dokumentiert sein [5].

## Versorgungslücken bei einer vulnerablen Zielgruppe

Menschen mit Depressionen sind durch Symptome wie Antriebslosigkeit, sozialem Rückzug und Bewegungsmangel besonders häufig von Risikofaktoren für Vitamin-D-Mangel betroffen.

Während bei der Allgemeinbevölkerung unspezifische Symptome wie Wintermüdigkeit oft Anlass zu Diagnostik und einer Supplementation sind, werden sie bei Patienten mit Depression oft primär der Grunderkrankung zugerechnet. Dadurch bleibt die Differenzialdiagnostik hinsichtlich einer Unterversorgung mit Vitamin D häufig aus – eine implizite Benachteiligung einer verletzlichen Patientengruppe. Für die Betroffenen bedeutet dies auch einen Verlust an Selbstwirksamkeitserleben, wenn ihre Handlungsmöglichkeiten auf die Rolle „Patientinnen und Patienten mit Depression“ verengt werden und mögliche, gleichzeitig bestehende somatische Ursachen wie ein Vitamin-D-Mangel in den Hintergrund treten. Dies sollte insbesondere beim gleichzeitigen Bestehen weiterer Risikofaktoren (höheres Alter, wenig Aufenthalt im Freien, dunklere Hautfarbe) zur Diagnostik bewegen.

Vor diesem Hintergrund erscheint es medizinisch inkonsequent und ethisch problematisch, wenn ausgerechnet für diese Hochrisikogruppe auf eine konsequente Überprüfung des Versorgungsstatus verzichtet wird, anstatt – analog zur Prophylaxe bei anderen Personengruppen – einen nachgewiesenen Mangel gezielt therapeutisch anzugehen.

## Diskussion und Fazit

Die NVL macht deutlich, dass Nahrungsergänzungsmittel keine Ersatztherapie für evidenzbasierte Behandlungsmethoden darstellen dürfen und warnt vor einer Verdrängung dieser durch eine vermeintlich „einfache“ Supplementation. Gleichzeitig kann eine gezielte Vitamin-D-Substitution als additive, sekundär präventive oder adjuvante Maßnahme bei gesichertem oder wahrscheinlich vorliegendem Mangel sinnvoll sein.

Die Realität zeigt, dass die Ernährungssituation bei Patientinnen und Patienten mit Depression oft unzureichend ist [10]. Psychosoziale Belastungen, mangelnde Alltagsstruktur und niedrige Ressourcen begünstigen eine inadäquate Vitamin-D-Versorgung.

Die NVL-Empfehlungen sind differenziert zu verstehen: Sie fordern ein diagnostikgestütztes, individualisiertes Vorgehen, das eine Substitution bei Mangel einschließt, ohne die Prävention von Mangelzuständen bei Risikogruppen durch Ernährungsinterventionen aus dem Blick zu verlieren.

Patientinnen und Patienten mit Depression sollten zumindest den gleichen Zugang zu Diagnostik und adäquater Versorgung haben wie nicht erkrankte Personen. Die enge Abstimmung ernährungsbezogener Empfehlungen in der psychiatrischen Versorgung mit den Leitlinien der DGE sowie der Einbezug von Fachgesellschaften aus dem Bereich der Ernährungsmedizin/Ernährungstherapie sind daher essenziell, um zukünftig widersprüchliche Empfehlungen zu vermeiden und bestehende Versorgungslücken – insbesondere bei diesen vulnerablen Patientinnen und Patienten – zu schließen.

## Fazit für die Praxis

- Unzureichende Vitamin-D-Versorgung ist im Herbst und Winter in Mitteleuropa weit verbreitet und betrifft auch Menschen mit Depressionen.
- Menschen mit psychischen Erkrankungen sollten denselben Zugang zu Diagnostik und Therapie erhalten wie die Allgemeinbevölkerung.
- Die Abstimmung der Empfehlungen zwischen nationalen Leitlinien, Fachgesellschaften und Ernährungsmedizin ist essenziell, um Versorgungslücken zu schließen.

## Data Availability

Alle dieser Arbeit zugrunde liegenden Daten sind in diesem Artikel enthalten.
